# Barriers and Facilitators to the Implementation of Virtual Reality as a Pain Management Modality in Academic, Community, and Safety-Net Settings: Qualitative Analysis

**DOI:** 10.2196/26623

**Published:** 2021-09-22

**Authors:** Urmimala Sarkar, Jane E Lee, Kim H Nguyen, Sarah Lisker, Courtney R Lyles

**Affiliations:** 1 Department of Medicine University of California San Francisco San Francisco, CA United States; 2 Center for Vulnerable Populations University of California San Francisco San Francisco, CA United States; 3 Department of Epidemiology and Biostatistics University of California San Francisco San Francisco, CA United States

**Keywords:** virtual reality, medical informatics, information technology, implementation science, qualitative research

## Abstract

**Background:**

Prior studies have shown that virtual reality (VR) is an efficacious treatment modality for opioid-sparing pain management. However, the majority of these studies were conducted among primarily White, relatively advantaged populations and in well-resourced settings.

**Objective:**

We conducted a qualitative, theory-informed implementation science study to assess the readiness for VR in safety-net settings.

**Methods:**

Using the theoretical lens of the Consolidated Framework for Implementation Research (CFIR) framework, we conducted semistructured interviews with current VR users and nonusers based in safety-net health systems (n=15). We investigated barriers and facilitators to a commercially available, previously validated VR technology platform AppliedVR (Los Angeles, CA, USA). We used deductive qualitative analysis using the overarching domains of the CFIR framework and performed open, inductive coding to identify specific themes within each domain.

**Results:**

Interviewees deemed the VR intervention to be useful, scalable, and an appealing alternative to existing pain management approaches. Both users and nonusers identified a lack of reimbursement for VR as a significant challenge for adoption. Current users cited positive patient feedback, but safety-net stakeholders voiced concern that existing VR content may not be relevant or appealing to diverse patients. All respondents acknowledged the challenge of integrating and maintaining VR in current pain management workflows across a range of clinical settings, and this adoption challenge was particularly acute, given resource and staffing constraints in safety-net settings.

**Conclusions:**

VR for pain management holds interest for frontline pain management clinicians and leadership in safety-net health settings but will require significant tailoring and adaption to address the needs of diverse populations. Integration into complex workflows for pain management is a significant barrier to adoption, and participants cited structural cost and reimbursement concerns as impediments to initial implementation and scaling of VR use.

## Introduction

Immersive virtual reality (VR) has emerged as an efficacious treatment modality for a wide range of medical and neurocognitive conditions including pain. VR is delivered via a headset that displays computer-generated and oftentimes interactive audio and visual content that can be designed to reduce pain through techniques such as distraction, relaxation, and mindfulness. Randomized controlled trials and effectiveness studies demonstrate that VR improves pain scores among inpatients and during medical procedures [1-4]. Additionally, studies indicate that patients can use VR to help manage chronic pain [5-8]. In a prior study among predominantly non-Hispanic White patients, most patients found VR use for pain to be a positive experience [9]. VR is generally considered safe, with the most common adverse effects being dizziness and nausea. Motion sickness and seizure disorders are the most prevalent of the few medical contraindications to VR use.

Clinicians have demonstrated interest in VR therapy as a safe and effective adjunctive or replacement to opioid agents to avoid adverse consequences of opioid use [10]. Opioid misuse represents a growing epidemic in the United States, affecting 10.3 million people, 48,000 of whom died in 2018. Even in acute-care settings, initiation of opioids can lead to long-term use, the consequences of which include dependence and hyperalgesia [11]. There is an urgent need for safer pain management for the millions of chronic pain sufferers worldwide. This represents a public health challenge as well, as racial and ethnic minorities, the elderly, and patients who speak English as a second language are more likely to experience suboptimal treatment for their chronic pain [12].

Despite studies proving the efficacy of and great promise for VR therapy as a nonpharmacologic approach to pain management, VR has not become part of routine chronic pain management [2,13,14]. As with other complex interventions, there is little evidence to guide implementation [15]. Although we know that VR has been effective in clinical trial settings, understanding why and how it has been effective is required to translate this modality to diverse practice settings [16]. Most VR studies have also been conducted in settings that serve ethnically homogenous, relatively advantaged populations with high health literacy and educational attainment.

There are specific considerations in adapting digital innovations to diverse populations and to safety-net health care settings that disproportionately care for them [17]. A systematic review of digital innovations in the safety net found that externally developed interventions face significant challenges, including acceptability to providers, staff, and patients; staffing needs; and implementation costs [18]. We sought to elucidate the implementation climate specifically for VR [19]. Therefore, we conducted a qualitative, theory-informed implementation science study to assess the readiness for VR in safety-net settings.

## Methods

This study aims to determine barriers and facilitators to the implementation of VR as a treatment for pain in safety-net settings. We obtained institutional review board approval (#19-29025) for this project from the University of California, San Francisco (UCSF) Human Research Protection Program.

### Conceptual Framework and Interview Guide

We developed an interview guide based on the Consolidated Framework for Implementation Research (CFIR) [20,21]. We chose this framework because it accounts for a wide range of contextual factors and is widely used to characterize complex interventions in health care settings [22-26]. The CFIR framework includes 5 overarching domains: (1) the characteristics of the intervention itself; (2) the inner setting, that is, the specific environment in which the intervention will take place; (3) the outer setting, or larger environment, encompassing the broader landscape in which the inner setting sits; (4) the individuals involved in intervention delivery and the target population; and (5) the process by which the intervention is introduced.

### Data Collection

We used the snowball method of sampling to conduct 15 semistructured interviews (Multimedia Appendix 1) of 5 health care providers, leaders, and administrators who currently provide VR to patients (users) and 10 who do not use VR (nonusers). Most but not all nonusers practiced in safety-net settings, defined as public hospitals or federally qualified health centers serving the majority of patients on Medicaid or those who are uninsured [27]. We intentionally sampled nonusers as health care providers or staff without experience in using VR for clinical pain treatment, ensuring that every participant had expertise working with diverse patient populations in their practice (i.e., safety net or academic medical center sites serving Medicaid or racially/ethnically or linguistically diverse patients). We oversampled safety-net sites in particular because they are generally the last to be considered for implementation of innovation programs, and we sought to seek out their perspectives early to improve eventual broadscale implementation of this technology for diverse patient populations [28]. We conducted interviews from October 2019 to September 2020. Two members of the study team conducted the interviews via videoconferencing with screen sharing because of either logistical barriers or public health restrictions due to the COVID-19 pandemic for meeting in person. The team recorded the interviews with participant consent and transcribed the interviews without any identifying information. The participants received an incentive for their participation, with e-gift cards sent via email following interview completion.

### Data Analysis

**Figure 1:**
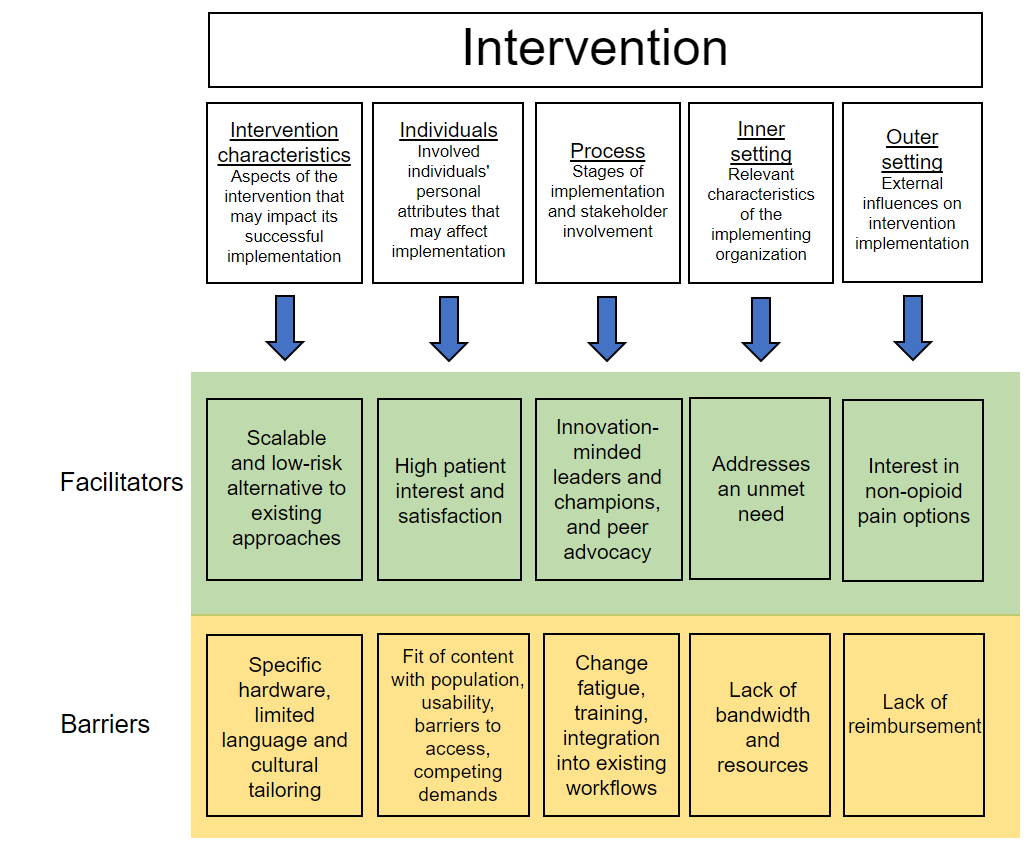
Overview of Consolidated Framework for Implementation Research (CFIR) domains and examples of barriers and facilitators.

We used the qualitative software Dedoose (SocioCultural Research Consultants, Hermosa Beach, CA, USA) for analysis. Two authors, KN and JL, read all the transcripts in detail. For deductive coding, they assigned excerpts to 1 of the 5 overarching CFIR domains (intervention characteristics, individual characteristics, implementation process, inner setting, and outer setting) and met to discuss codes and resolve inconsistencies (Figure 1). They then independently analyzed 3 interview transcripts inductively to identify themes that arose from the interviews. They subsequently met to reach an agreement on a comprehensive list of potential themes. One investigator (author JL) coded the remaining (12 total) transcripts under the supervision of KN for any relevant CFIR constructs and new inductive codes that emerged from participant responses. The entire study team met regularly to discuss discrepancies or resolve disputes regarding the coding. All authors iteratively revised and agreed upon the final list of themes and representative quotes. We reached thematic saturation after 11 interviews and elected to complete 4 additional interviews that had already been scheduled.

### Data Availability

The qualitative data generated and analyzed during the current study are not publicly available due to the risk of re-identification of participants, but codebooks are available upon reasonable request.

## Results

We interviewed 15 participants from a variety of roles and organizations (Table 1).

We defined “user” as a health care provider or staff member interviewee with previous research experience implementing the AppliedVR platform for pain treatment at their delivery system; we defined “nonuser” as a health care provider or staff member interviewee without experience with AppliedVR or other VR platforms for clinical pain treatment.

Underlying these results was a strong sense of an unmet need for chronic pain management among users and nonusers. There was agreement among all interviewees that addressing pain management differently represents a high organizational/clinical priority. Building on that universally acknowledged need and prioritization, both users and nonusers of VR identified a range of factors related to the intervention itself and to the milieu in which it would be implemented that they expected would impact VR implementation. We described the major themes within each CFIR domain and provided exemplar quotes.

**Table 1:** Participant roles, user status, and organization characteristics.

Participant ID	Participant role	User/nonuser	Organization characteristics
1	Chief Medical Information Officer	User	Safety-net health system
2	Research physician	User	Academic medical center
3	Clinical research coordinator	User	Academic medical center
4a	Director for Innovation and Digital Health^a^	User	Academic medical center
4b	Program Manager for Innovation and Digital Health^a^	User	Academic medical center
5	Director of surgical subspecialty service	User	Nonprofit regional tertiary medical center
6	Primary care and addiction services physician	Nonuser	Safety-net health system
7	Associate Director of Research and Health Equity	Nonuser	Academic medical center
8	Chief Medical Officer	Nonuser	Safety-net health system
9	Chief Medical Informatics Officer	Nonuser	Safety-net health system
10	Primary care physician, pain clinic	Nonuser	Safety-net health system
11	Director of Telehealth	Nonuser	Safety-net health system
12	Psychologist, internal medicine	Nonuser	Academic medical center
13	Registered nurse, palliative care	Nonuser	Safety-net health system
14	Associate Chief Medical Informatics Officer	Nonuser	Safety-net health system
15	Psychologist, internal medicine	Nonuser	Safety-net health system

^a^These participants were interviewed simultaneously.

### Characteristics of the Intervention

The VR intervention itself has attributes that both facilitate and inhibit implementation (Multimedia Appendix 2, Table S1). A key factor for the intervention is its relative advantage over existing approaches for pain. VR users and nonusers both mentioned the favorable safety of using VR compared to pain medications. Additionally, VR users also mentioned the option for patients to use the technology on their own on an ongoing basis after initial training from staff, as opposed to ongoing, clinician-delivered pain interventions, such as cognitive behavioral therapy. Similarly, nonusers appreciated the potential for scaling pain management activities through VR-enabled self-management approaches. Nonusers of VR commented on the face validity of VR as a chronic pain treatment. In terms of the intervention content, both users and nonusers noted that the platform is available only in English and therefore not usable in limited English–proficiency populations. The difference between users and nonusers was in how they prioritized this limitation. Interviewees based in the safety net described non-English language as a prerequisite, whereas it was considered a limitation of varying importance among those who were already using VR. The participants discussed the ability to culturally tailor VR scenarios in opposing ways. VR nonusers expressed some skepticism about the content’s appropriateness for culturally diverse populations and for those with significant trauma histories. VR users noted that the currently available content does not always resonate across cultures, particularly for minoritized populations. However, they did consider the ability of VR to offer many culturally tailored content offerings to be a key attribute of the approach. Both users and nonusers acknowledged the specific contraindication of motion sickness and the overall low risk of the modality.

### Individual Characteristics

For this study, we considered the CFIR domain of “individual characteristics” to refer to target patients for VR as a pain intervention (Multimedia Appendix 2, Table S2). A prevalent theme was comfort with technology, including challenges for older adults in navigating technology and digital literacy more broadly. Nonusers, in particular, raised concerns about patient mistrust of new or experimental treatments. Despite this, several interviewees emphasized that they would expect a significant proportion of low-income and diverse patients cared for in safety-net settings to willingly engage with VR. Participants who used VR expressed near-universal high levels of patient satisfaction with VR, and nonusers expected that patients with chronic pain would appreciate having additional treatment options. Nonusers did cite physical opioid dependence and a fear of opioid de-prescribing as expected barriers from patients, and users and nonusers alike referred to patients’ more pressing social and health needs as significant barriers to VR implementation for pain. Nonusers specifically mentioned trade-offs for health systems in deciding whether to invest in innovations such as VR versus approaches to address patient needs such as housing and food insecurity.

### Implementation Process

Both VR users and nonusers considered VR implementation to be a complex process (Multimedia Appendix 2, Table S3). There was consensus that patients would require specific orientation from staff in order to initiate VR use for pain and that staff support would be required for coaching and troubleshooting on an ongoing basis. Both users and nonusers pointed to the requirement that frontline staff interact with VR and have a personal buy-in to facilitate successful implementation. Similarly, all participants highlighted the need for champions among clinicians who can share both evidence for VR as a pain treatment and successful treatment experiences. The availability of staff time was a dominant concern, with VR users emphasizing the need to plan for adequate staff effort to implement VR and nonusers expressing doubt about staff availability. Integration of VR into existing pain management workflows, a key aspect of the implementation process, highlighted barriers and facilitators. It emerged that implementation success would depend on the extent to which VR could be integrated into specific clinical settings. Some, but not all, nonusers focused on integration into the electronic health record. The participants cited some especially challenging settings, such as primary care, and some more feasible settings, such as the outpatient pain clinic. More specifically, the intervention requires a multistep process with each patient, which both users and nonusers believe complicates more widespread implementation.

### Inner Setting

The inner setting for VR implementation is the specific health system and, within each health system, the specific clinical settings that currently treat pain (Multimedia Appendix 2, Table S4). One overarching inner setting barrier, seen across all participants, is that within health systems, pain management is a cross-cutting issue and therefore requires collaboration among departments and stakeholders, in contrast to other clinical conditions for which one clinical unit is entirely responsible. Both users and nonusers cited pressure to prescribe opioids and the providers’ lack of familiarity with VR as barriers to use. Several participants mentioned the default habit and culture of addressing pain with medication. Among the nonusers, participants noted that their peers hold skepticism about corporate approaches to treatment. Both users and nonusers emphasized the importance of leadership attitudes. They described interest in VR as part of a larger mindset around openness to innovation. All participants emphasized the need for dedicated staff time and training and space for VR treatment, which require a leadership commitment. Resource limitations in safety-net settings were seen to preclude these needed actions for implementation. Most participants suggested that an innovative culture is a prerequisite for VR implementation, but safety-net participants described this as pervasive: “We seem to be late adopters.” In contrast, VR users described few individuals as having an anti-innovation attitude. Some nonusers described local leaders as not being innovation minded but instead being focused on delivery of long-standing services. Similarly, nonusers described the wide array of stakeholders (eg, frontline staff, information technology [IT], and clinical leaders) within the local environment whose engagement was needed for success. In terms of concrete needs, both users and nonusers cited the need for adequate physical space for VR treatment and up-front investment in the VR hardware as inner-setting requirements. However, VR users experienced these needs as surmountable challenges, while VR nonusers highlighted space and initial costs as formidable obstacles.

### Outer Setting

Challenges related to the outer setting of the intervention, such as external policies and incentives, arose as well (Multimedia Appendix 2, Table S5). In the broader health care landscape, use of VR is not a billable service. The participants viewed this lack of insurance reimbursement as a critical barrier to implementation at scale. Both VR users and nonusers mentioned philanthropic and private funding as a current or possible approach for the short term. There was consensus that expecting patients to bear the cost of pain treatment is a significant implementation barrier. Beyond cost, concerns around data privacy and security in digital health surfaced among nonusers. Nonusers also pinpointed the digital health industry’s focus on wellness over developing treatments for specific conditions as a barrier to widespread use of digital therapies more broadly.

## Discussion

### Principal Results

Our results suggest that many of the factors affecting VR implementation, or, indeed, implementation of novel, technology-enabled therapeutic approaches, exist in both safety-net settings and more advantaged, resource-rich health care systems. We did not identify a completely different set of issues relating to VR in the safety net; rather, similar challenges seem to be magnified in safety-net settings. For example, among safety-net stakeholders who do not currently use VR, an English-only intervention would be impossible to implement for their linguistically diverse patient populations, whereas non-safety-net participants saw this limitation as less significant in impacting adoption. Similarly, the cultural relevance and acceptability of the content was a source of concern for safety-net interview participants but appeared to be surmountable to non-safety-net participants who were already testing VR at their sites. In other words, current VR users saw further cultural tailoring as an area for future improvement, whereas our safety-net participants saw it as a prerequisite for use because of the diverse populations served in safety-net settings. Both VR-use and VR-nonuse sites acknowledged that competing demands in patients’ lives, relating to housing status, income instability, and other social challenges, would interfere with using a novel therapeutic approach like VR. Again, these challenges seemed more prevalent in the safety net.

Our findings in the CFIR domain of “inner setting” suggest that the process of implementing a complex intervention like VR requires a formal change management strategy encompassing leadership support, frontline champions, and a campaign for staff buy-in. Safety-net respondents reported a lack of capacity for implementing new approaches, particularly with regard to leadership commitment to innovation and resources to conduct change management activities. The outer setting or larger context for VR implementation has facilitators, such as the desire to appear innovative, while reducing the harm from opioid use. However, the lack of insurance reimbursement and available funding for VR interventions is a significant barrier. Competing priorities for leadership can also stymie innovations such as VR implementation, especially in safety-net health care settings where resource limitations and “keeping the doors open” are perennially in question.

Our findings echo prior work about innovation in safety-net settings. Dating back to the early days of health IT, safety-net settings have lagged in implementation [29]. Prior studies suggest that frontline patient engagement and trust, appropriateness of innovative tools for diverse end users, and dedicated resources for innovation are particularly salient for the implementation of new health IT approaches in the safety net [18,28,30,31]. Implementation science studies in safety-net settings should specifically emphasize these concepts in applying, and, where necessary, adapting, conceptual frameworks such as the CFIR.

Our study lends weight to a previously developed evaluation framework for VR [19]. The VR1 study design phase emphasizes the importance of working directly with end users, and we found that implementation in safety-net health settings will require significant tailoring and adaptation of the VR intervention to address the needs of diverse populations (eg, racial and ethnic minorities, language barriers, and social complexity). Several actions can address the current barriers to VR use for pain management in safety-net health care settings. For example, our results underscore the need for cultural tailoring and translation (eg, VR content that features voices and images of racially and ethnically diverse individuals and is provided in languages other than English). Furthermore, more specific adaptations or considerations addressing structural issues disproportionately facing lower-income and racial/ethnic minorities in the United States (eg, unjust incarceration practices and historical trauma) might require deeper qualitative exploration with patients about their preferences or comfort in using VR headsets. A prior study of predominantly non-Hispanic White hospitalized patients demonstrated that many refuse to try VR [9]; the acceptability of VR to patients should be tested in more diverse patient groups. Following this, usability testing with diverse populations could evaluate concerns about the acceptability and usability of technology, disinfection, and adverse effects such as motion sickness. Any deployment strategy for VR should take into account the resource and workforce constraints of the safety-net environment. Developers of VR for chronic pain management should address implementation considerations in parallel with design and evaluation in order to foster higher uptake of VR and digital tools overall.

### Strengths and Limitations

Our study has strengths, such as inclusion of multiple health systems and geographic areas. In addition, we compared and contrasted the experiences of health care system participants who are currently overseeing the use of a single VR platform with those of safety-net and academic providers across multiple sites who are not currently using VR. Furthermore, we used a well-known conceptual framework and reached thematic saturation in content analysis. Despite these strengths, the study does have limitations. The sample size was circumscribed, and we do not have information about the extent of VR use among the participants who are currently using the tool in their health systems.

Although our discussion focused on one technology approach, VR, for a single clinical problem (pain management), many of the themes that emerged are relevant across multiple innovative approaches to clinical care. The spread of innovations to safety-net health settings requires attention to patients’ language and culture. Efforts to address resource constraints, such as providing staff training and detailed protocols encompassing workflow integration, can lower barriers to innovation. Inclusion of diverse populations and the health care settings where they disproportionately receive care will address issues that are relevant across other health systems as well.

### Conclusion

This study contributes to the literature by revealing barriers and facilitators to implementing VR for pain management in safety-net settings. We demonstrate that frontline pain management clinicians and leaderships are interested in VR, but it will require significant tailoring and adaptation to address the specific needs of the diverse populations they serve. The participants cited integration into complex workflows, structural costs, and reimbursement concerns as major barriers to implementing and scaling VR use. Future studies should augment this approach with direct observation, quantitative measures of VR use, and direct usability assessment of the AppliedVR platform with diverse patient populations.

## Appendix

Multimedia Appendix 1 Consolidated Framework for Implementation Research (CFIR) structured interview guides for users and nonusers.

Multimedia Appendix 2 Participant quotes.

## Abbreviations

CFIR: Consolidated Framework for Implementation Research
IT: information technology
UCSF: University of California, San Francisco
VR: virtual reality
