# Supplementary material for: Barriers and Facilitators to the Implementation of Virtual Reality as a Pain Management Modality in Academic, Community, and Safety-Net Settings: Qualitative Analysis
Source: J Med Internet Res. 2021 Sep 22;23(9):e26623. doi: 10.2196/26623 (PMC8495579; doi:10.2196/26623)
Supplement: Multimedia Appendix 1 [file jmir_v23i9e26623_app1.docx]

Appendix 1. CFIR structured Interview Guides for users and nonusers.

Interview Guide

Current users

Interviewee and organization information

1. Please describe your role (related to pain management approaches) and how long you’ve worked in it.
2. Can you outline in broad terms the current pain management programs that exist in:
   1. Post-surgery
   2. Peri-operatively
   3. During treatment
   4. Outpatient care
   5. Overall

Intervention characteristics and process for implementation/ current engagement with digital health tools:

1. Walk me through your workflow using AppliedVR technology. Who assisted patients with using it?
2. What were your metrics for success? For patients, doctors, nurses, etc.?
3. What type of pre-implementation training was needed? How long did it take? Were nurses (or whoever was using it) compensated to learn to use it?
4. [If the system was offered as a part of ongoing care] How much staff time did it take to use? From whom? Was it available 24/7 or was it more limited?
5. How was it offered to patients? Do you have a sense of what proportion of patients who were offered it actually tried it? Were there certain patient populations that didn’t want to/ couldn’t use it? What barriers do you think they faced?
   1. For patients that were onboarded, what proportion dropped out? Why do you think they dropped out (e.g. the technology itself not a good fit, or something else?)
6. How did the use of AppliedVR within this clinic/unit impact your usual, daily work?
7. Are there components of AppliedVR that should be altered? That shouldn’t?
8. What changes did you make to the protocol you planned?
9. Now that your study is over, what are the plans to continue/ expand use of VR?
   1. If not currently feasible, what do you think it would take to become feasible?
10. What costs were incurred when implementing? Are there any additional resources you needed to support the implementation?

Internal factors

1. How did the AppliedVR intervention compare to other similar existing programs in your setting? What about to alternatives that have been considered? Is there something else people would rather implement?
2. How well did the intervention fit with your organization’s norms and practices?
3. Where did you have workarounds or improvise?
4. How did you document VR in the electronic health record? Were any infrastructure changes required?

External factors

1. What policies/ incentives do you anticipate being instrumental in the future success of this work?
2. What challenges do you anticipate related to scaling? What about facilitators to scaling?

Characteristics of individuals

1. We are interested in people’s reactions to the idea of using VR. What did health care team members (assistants, nurses) say? Doctors? Leadership?
2. Were there people at your site (or outside) that are particularly important in implementing digital health? What did it take to get them engaged in the VR implementation?
3. Any skeptics? How did or will you handle them?
4. Have you elicited information from patients regarding their experiences with the intervention?

Interview Guide

Nonusers

Interviewee and organization information

1. Please describe your role and how long you have worked in it
2. Please tell us about your organization (if applicable)
   1. Years in operation
   2. Mission/ goal
3. What is your role in terms of the pain management approaches that are used in your organization?
4. Can you outline in broad terms the current pain management programs at your organization in: (post-surgery only? Outpatient care? Overall?)
5. Have you heard of virtual reality to manage pain? What are your thoughts about this?
6. Have you ever used virtual reality before? What was the setting?

External factors

1. Are there people at your facility who have been particularly important in helping implement digital health? What would it take to get them engaged in an implementation of VR?
2. What policies/ incentives have been/ do you expect to be instrumental in the success of a VR tool?
3. What challenges do you anticipate related to implementing VR, or scaling? What about the facilitators to scaling?
4. To what extent might implementing VR provide an advantage to your organization compared to other organizations in your area?

Internal factors

1. Is there a strong need for pain management interventions? How do people feel about existing programs?
2. How might needs for VR be different at your site than elsewhere?
3. How do you think your organization’s culture might affect implementation of a digital health tool using VR?
4. Would implementing VR require a change to your workflow?
5. What other interventions might you bundle with it?
6. What are your requirements for integrating/ accessing new technologies, such as VR, in the electronic health record?
7. Do you currently have sufficient resources to support digital health implementation? A specific VR implementation?
   1. What resources would you need to do so, if not? What infrastructure changes might be needed?

Intervention characteristics and process for implementation

1. If you were to use a VR tool in clinical care, what level of evidence would you expect beforehand? What would be your metrics for success? Why might you choose VR for pain management over an alternative?
2. What would you need to know in order to think it was worth it?
3. What would you need in order to feel comfortable recommending VR treatment?
4. What types of implementation or other operational challenges might you anticipate encountering? Are there implementation leaders or champions that might go above and beyond to implement a tool like VR?

Characteristics of individuals

1. How would you feel about an intervention like VR being implemented in your setting? Do you think it would be effective? What do you think your colleagues would think?
2. What do you think your patients would think of VR for pain management? How might this vary by patient characteristics (e.g. race, ethnicity, socioeconomic status)?
   1. PROBE: Are there certain segments of the population you’d be less likely to consider for VR treatment?
