# Supplementary material for: Barriers and Facilitators to the Implementation of Virtual Reality as a Pain Management Modality in Academic, Community, and Safety-Net Settings: Qualitative Analysis
Source: J Med Internet Res. 2021 Sep 22;23(9):e26623. doi: 10.2196/26623 (PMC8495579; doi:10.2196/26623)
Supplement: Multimedia Appendix 2 [file jmir_v23i9e26623_app2.docx]

Appendix 2: Participant quotes.

Table S1. Intervention Characteristics

| CFIR Domain: Intervention | | |
| --- | --- | --- |
| *Theme* | *User sub-theme,* quote | *Nonuser sub-theme,* quote |
| Alternative to pain medications | *VR is an appealing alternative to pain medication*  “People would say they would use it to avoid using pain medication because let’s say it was in the middle of the day and they couldn’t take their medication because it made them drowsy, they would use VR instead.” – Participant (Ppt) 3  “Participants reporting good things about using VR such as distraction from pain, anxiety reduction, alternative to pain medication kind of thing, I mentioned like facilitators.” – Ppt 3 | *VR is safe*  “I don’t think this is anything that introduces potential patient harm.” – Ppt 6  *Need for low-cost pain interventions*  “The answer is there are currently none [no patient-facing tech intervention]. So, there are providers that may recommend apps to certain patients, but that’s a very individualized kind of decision. The barrier with some of the just thinking about apps is “Does the patient facing technology is that some of them had a cost?” so that’s a barrier.” – Ppt 6  *Lack of non-pharmacological pain management options*  “I think particularly in a low resource setting where patients haven’t had options, they haven’t had any kind of behavioral or alternatives of pain management methods and for the most part, they’ve never seen a psychologist or been introduced to any of these concepts whatsoever.” – Ppt 12  “So, we’ve got kind of a captive audience, right? So, in a way, wouldn’t it be amazing if like, “You know, [name], instead of giving you 120 of methadone a day, I’m going to put this headset on while you’re getting your infusion and let’s see what we can do.’” – Ppt 13  *Can exacerbate isolation*  “It’s a solitary activity and sometimes that’s healing for people with chronic pain and sometimes not because there’s already lots of a solution for people experiencing pain like they feel very alone and that people don’t understand them. That’s a big part of what sort of exacerbates their pain.” – Ppt 6 |
| Pain management is a high priority | *Need new solutions to address pain*  “We have several pain clinics and the people there – one was the psychiatrist with the pain clinic, another was the pain clinic director who’s a psychologist, and so one of the other pain clinic staff who I think was also a psychologist, and they just looked at the different solutions and I think they chose it just because they thought that it seemed to have the best applicability to our patients, that it seemed to have the most relevance to treating pain.” – Ppt 1  *Gaps in current treatment exist*  So, pain management is a scenario where everybody can benefit from improving quality because we know in general that pain is not perfectly treated.” – Ppt 2 | *Desire for non-medication options*  “I think us being affiliated with an academic institution, we’re always very curious to learn about new innovations and the new technologies that could help our patient and especially in a topic such as pain control when we’re trying to find non-medication options for patients. So, I think there would be an appetite for it and people would definitely be curious in testing it and using it.” – Ppt 8  *Untreated pain is prevalent*  “I feel like there’s a lot of suffering out there, and if people can . . . go through your normal treatment algorithms and still can’t meet those functional goals, then it’s important to be a good steward and to try alternatives” – Ppt 14  *Chronic pain is isolating*  “I think that the social aspects for chronic pain regardless of your symptoms and economic status, if you’re experiencing chronic pain, it’s hard to motivate. It’s hard to get out, interact with family members, and participate of getting a social life. I think that that is exacerbated for vulnerable populations in terms of just not having the same level of resources to help fill some of those gaps.” Ppt 7 |
| Face validity of VR | “I’ve had phone calls from a nurse champion who does the naturopathic service consult service at a [state] hospital. She was in a phone call with a bunch of MDs trying to do a big study and she was like, ‘Oh, I don’t need a big budget. I’m just trying to do it myself.’ She’s wild. She’s awesome. She’s not telling me about her FAs and her indirect. She’s just like, ‘No, just give it to me.’” – Ppt 2  “I’m the chief medical information officer here and I’ve been helping to lead innovation at the medical center, and the reason that I’ve gotten involved was I had discovered virtual reality was used for chronic pain management and I reached out to our pain clinic director and she actually luckily had already seen a lecture on virtual reality and so she was already prompted and willing to go for it, and so that’s how that sort of started.” - Ppt 1 | “If I want to do a distraction like I want the patient to do some sort of distraction technique, VR seems to be like – I’ve never tried VR but it seems to really take the patient into that space and distract them from their pain. Usually when I’m working with a patient, I’m doing these mindfulness pleasant imagery types of exercises. If you’re using VR – correct me if I’m wrong about any of this – I would imagine that they can really immerse themselves in that and I mean it’s still them doing work but it’s less work to get them there because they’re immersed in the virtual reality experience.” – Ppt 15 |
| VR technology can scale existing pain management approaches | “So, breathing exercises, meditation was number one. It helped, within the VR, doing the breathing exercises but then outside a lot of patients after maybe two weeks of using VR, they were like, ‘Yes. What I did was close my eyes and just imagined I was back in doing the VR again and then just practice the breathing exercises. Using what the VR taught me, I was doing it outside of that.’” – Ppt 3 | “But yes, [I would be] definitely able to either sit back, watch the patient, observe the patient, take that time out, and not have to actually do the distraction exercise with them.” – Ppt 15 |
| Non-English language VR availability | *Few limited English proficiency patients*  “It’s not much of a barrier because the percentage of the patients that we have that are non-English speaking is less than 10%. They’re probably at 5%.”- Ppt 5  *Lack of access for Spanish*  *“*AppliedVR doesn’t have anything in Spanish and as you can imagine, as you know in LA, there’s a fair amount of Hispanic patients. Not having an intervention that can speak that language, it’s pretty hard to get them interested. So, there’s a language difference*.”* – Ppt 2 | *Available in different languages*  “Some of these health systems [that use VR], it’s not really what our patients are*. I don’t know if these [VR approaches] are in different languages.” -* Ppt 6  *Language and healthy literacy as barriers*  “… our patients have very low health literacy in many respects. Also, we have multiple languages that our patients speak or English is not their primary language. So, I want to be sure that when we implement something that those things are taken into account and those things – potentially, those barriers could be overcome for our patient population so they could actually have access to the device or the technology.” - Ppt 8 |
| Cultural tailoring | *Not culturally tailored*  “The narration doesn’t necessarily resonate with them because I guess from their standpoint, they can tell it’s not someone that they relate to. Yes. ‘A Caucasian female who’s telling me how it is and telling me about this meditation stuff, and I’m just not buying it.’” – Ppt 2 | *Cultural tailoring*  “I think there are some differences on our patient populations… I don’t know like there’s questions about cultural concordance. I don’t know if my patients from Inland China want to watch an instant escape module about a beach in Hawaii. I don’t know. Maybe they do. [Laughter] Is that their version of escapism? I don’t actually know the answer to that?” – Ppt 6  *Ability to tailor*  *“*People definitely feel a lot more connected and invested if they were able to make a change to whatever you presented, you would be able to insist, ‘Oh, I didn’t really like the forest scene. It would be much better if it was like a rainforest in Puerto Rico rather than like a forest in Northern Canada,’ or something like that*.”* – Ppt 12  *May not account for trauma-based history*  “For safety-net patients, for things like guided imagery or certain types of visualizations, even breathing exercises, I would be cautious about the types of examples that are used. It’s like nature for some people. It’s their safe place. That’s where they’re inspired and calmed. For other people, it’s incredibly triggering. So, different aspects of how people have potentially experienced trauma in the past and what those triggers are can be a little bit unpredictable. So, I think with something like VR where there are specific templates, I think that would be one of my cautions just thinking about how can trauma informed care and that flexibility with how people can adapt to certain modules or templates, but that would certainly be part of my considerations.” - Ppt 7 |
| Limitations of content and use cases | *Limitations of content*  I think the ability to create the device to be more consumer/patient initiated, *and then to curate content specific to different types of healthcare interaction* would be [needed].” - Ppt 4  *Content not engaging*  “…so I came up with a theme of simulation homogeneity, just pretty much all the modules, they felt there was pain to them so they just got bored of it.” – Ppt 3 | *Need for more use cases*  “I think the use cases will come. If you get people comfortable with what it does and what it can do then that’s where you leverage really the innovative minds of providers themselves to come up with those use cases.” – Ppt 11 |
| Physical device considerations | *Bulky headset*  “One of the things was the bulkiness of the VR headset was the issue for mainly people - they tried it on the first week and then they were like, “I can’t do this. It’s too bulky.” – Ppt 3  *Ease of use*  “To get something new like this that is relatively easy to use, doesn’t really disrupt their workflow too much, the patients like it, you can see results...” – Ppt 1  *Need for hygiene*  “We had to come up with one-size-fits-all products for cleaning of the units between patients. We chose the [cleaning product] which is a UV sterilization unit. What we’re currently working on is building a cart which… basically has a battery pack on the floor. It has the [cleaning product] unit like it’s built into it and then it has the lock at the top of the unit… the different headsets, some of the long strap that can’t be wiped. Some of them have another strap which can be wiped down with the steady wipes. Then the inside lens, you can’t use any of that stuff. You have to use this particular lens spray to mop through those.” – Ppt 5 | *Cost of hardware*  “… it’s cost, hardware.” – Ppt 11 |
| Potential of seizures, motion sickness, or vertigo | *Higher levels of cyber sickness than reported in literature*  “About 4.7% of the participants reported ongoing side effects. Every time they would use it like within a few minutes, they would get dizzy and they would have to stop… it was a little bit more than what’s reported in the literature. The literature is like 1% or 2% or something like that.” – Ppt 3  *Motion sickness is rare*  “There’s other kind of stop relative to contraindications, people with a significant issue of motion sickness or vertigo. Although we allow those patients to still try it out, very rarely we have problem with patients discontinuing them if they have motion sickness.” – Ppt 5  *Should not be offered to patients with motion sickness*  “No, they have had it – in plans of the device, if they have nausea, if they have vertigo, there’s quite a long list of exclusion criteria that the company provides.” – Ppt 4 | “Again, side effects such as motion sickness and other things that I would want to know.” – Ppt 11 |

| Table S2. Individual Patient Characteristics | | |
| --- | --- | --- |
| CFIR Domain: Individuals | | |
| *Theme* | *User sub-theme,* quote | *Nonuser sub-theme,* quote |
| Comfort with technology | *Comfort with technology*  “It didn’t work 100% of the time. It seemed to work better on younger patients rather than older, and I think that has to do with familiarity or openness to technology...” – Ppt 1  *More difficult for older patients*  “It was like age, like a generational issue. Again, I don’t know the percentages but the patients I spoke to who I would say about 70 years old, it was a little bit difficult for them to use VR. They would have technical issues, we’ve resolved it and then on the next call it wasn’t necessarily a technical issue anymore but it was just, ‘… I could use it. I know how to use it but I just don’t want to use it because I just feel even doing this every time is just too hard.’” – Ppt 3  *Discomfort with technology even with access*  “Then there was other things that came up within that group where emailing was difficult, like answering the questionnaires was difficult. It wasn’t just like the VR, it was like everything in it was almost - a lot of the stuff that has to do with technology or anything like that, it was a little bit more difficult. They would complete it but it always took a little bit more to do it... The amount it took for them to do it really made them lose their motivation to even do it.” - Ppt 3 | *More difficult for older patients*  “I think there’s probably an age and sort of technology comfort thing that is there as well. I can see sort older patients maybe just needing a bit more support on kind of what this is, and how it works, and that type of thing.” – Ppt 6  *Patients lack of digital literacy*  “I don’t know. I think part of the unknown is I can’t even tell you how many of my patients have a smartphone or internet or a computer or restricted [data] plan. People’s tech literacy like never ceases to surprise me.” – Ppt 14  *Discomfort with technology even with access*  “Some of the more marginally-housed patients who don’t really have phones that are appropriate, or they have a very tight data plan or something. In this day and age, most people have unlimited data and Wi-Fi and a phone, so why they won’t accept it, I really, really don’t know.” – Ppt 13 |
| Mistrust |  | *Mistrust*  “Then, of course, you have to sort of get around the issue of just like, “Are you experimenting on me,” and sort of the typical mistrust thing. I think that there would be curiosity about it particularly if the provider said, “I think this might help you,” or if the provider is sort of saying like, ‘Why not try this?’ If that patient trusts the provider, that patient is more likely to try something new.” - Ppt 6  *Fear of “experimentation”*  “There’s also again, the stigma of being the guinea pig in our patient population. I still think that’s a common feeling amongst our patients or some of our patients. That means that you’re just testing on us to try to make some money without any real benefit to us.” – Ppt 11 |
| *Interest in VR or other innovative interventions* | *Patient satisfaction with VR*  “Yes, they say whenever a patient uses it, patients love it… the social workers are following the same patients that assist them through their care. So, they’ll see a patient multiple times and what they have gone back to me on is that those patients that have used it, they’re always asking for it the next time they see them.” – Ppt 4  “Patients are just blown away by it. They just feel like there will be – it makes them have a higher thinking of the hospital.” – Ppt 5 | *Willingness to try VR*  “I think with younger patients – let’s say you gave me one of these masks and then I took it to [pain clinic] and said, ‘Who wants to watch this mask thing and sort of relax and that type of thing for 30 minutes?’ I think a fair number of people would be like, ‘Oh, sounds cool.’” – Ppt 6  “I think if you choose the right patient, there will be many patients who are open to this. I’m pretty sure about this because I know the reaction to the pain group, the pain stuff. A number of them are enthusiastic.” – Ppt 10  *Excitement for innovative interventions*  “Yes, I think there’s no barrier. You can’t assume that lower socioeconomic status patients wouldn’t be enthusiastic about this. They would be.” – Ppt 10  *Patients appreciate being offered a treatment option*  “Just having options. I think particularly in a low resource setting where patients haven’t had options, they haven’t had any kind of behavioral or alternatives of pain management methods… patients just like this idea of like, ‘Oh, this is for me. This is for us... You made this available specifically for our group.’ I think that just that attention kind of increases morale; at least a positive effect and then positive aspect. Perceptions can lead to improved outcomes.” – Ppt 12  *Prevalent use of complementary alternative pain approaches*  “If you look at surveys or patients with chronic pain, the bulk are interested and already do complementary and alternative treatments for pain. So, it’s not that there’s a lack of interest.” – Ppt 6  *Enjoy using VR*  “So, I don’t think that it needs to actually improve patients’ behaviors or their pain outcomes directly but I think that as long as they enjoy using it… I think that’s great.” – Ppt 12 |
| *Opioid dependency* |  | *Opioid dependency*  “The main challenge is that patients are physically dependent on opioids most of the time that they’re taking them so long. The opioids, they realized, are not effective so sometimes that they have a little bit of cognitive flexibility in terms of a lead to the specific opioid is going to help them. They need to have it prescribed to them.”-Ppt 12  *Patient fear of getting opioids deprescribed*  “If they are getting these behavioral techniques, then the physician who believe that they no longer need to have these opioids so they were like in this kind of older – yes, this kind of rigid thinking of it’s either/or, black or white. . . in the short term, opioids are respected and they’ve been behaviorally conditioned to see it as being their effective way of managing their pain even though they complain that their pain isn’t being managed well.” – Ppt 12  “Initially, I was imagining little by little, like an EMDR [eye movement desensitization and reprocessing] session. It’d be like, and this is fantasy thinking, we’d put you in this room, this extra room that we have made with a couch and you put your feet up and we’re going to hook you up to this device. I also think that patients get so obsessed with opioids... If I could say, ‘Look, I’m going to call this company and tomorrow they’re going to deliver this device to you, and I want you to use it every day for a month and then let’s talk.’ So, not saying no, because there’s nothing going to alienate the patient faster than you saying, ‘I’m not going to give you your pain meds,’ If it’s like we work together on this, because not everybody wants to be as hooked on to the opioids as they are. We can rotate. We can try patches and we can try long acting, but at the end of the day, it’s all things that are just going to make you constipated and drowsy.” – Ppt 13 |
| *Stigma of mental health treatment* |  | *Stigma of mental health treatment.*  For me, particularly with low income, minorities who seen the psychologist or therapist of any type… it’s not in their typical lifestyle routine. Nobody in your family seen a psychologist. If somebody in the family saw a psychologist, they would be called crazy. The stigma associated with just behavioral vehicle or aside from anything even in the psych period. It’s just very challenging.” – Ppt 12 |
| Competing Demands | *More pressing unmet needs*  “We took patients from the trauma population. In trauma population, as you know, is very heterogeneous in their socioeconomic status. Those that I had, I can remember vividly at least a couple patients that withdrew that had a lot of issues with addiction and also housing status. They understandably – a few of them actually signed up for the study, but it was almost instant that we would lose all communication with them.” – Ppt 2 | *Other social barriers*  “It came up to a certain degree with the integrative pain management program all through other social factors in terms of what it takes to get to the clinic, the transportation challenges, getting on and off the bus versus somebody being able to drive you to the clinic, so those structural pieces that impact [Unintelligible] folks with chronic pain in a really different way.” – Ppt 7  *More pressing unmet needs*  “On the one hand, no, our patients don’t need VR. They need food stamps. My patient doesn’t have a safe place to sleep, let alone take-home goggles.” - Ppt14 |

Table S3. Implementation Process

| CFIR Domain: Implementation Process | | |
| --- | --- | --- |
| *Theme* | *User sub-theme,* quote | *Nonuser sub-theme,* quote |
| Demonstrating and offering VR to patients | *Need to explain rationale for VR use*  “I think in an outpatient setting where the person has the VR headset and let’s say… they’re VR naive. I don’t think in that group just giving them a VR headset alone is going to be efficacious. I think it does require, one, to talk about the benefits of VR with them, give them some basis of why they’re using it.” – Ppt 3 | *Peer advocacy of VR more convincing*  “Yes. It really is how it’s presented, 100%...The other thing that we’re really exploring is how about having another patient come in and say, ‘Hey, I’ve had chronic pain. I’ve been trying out this VR headset thing. I know what it’s like to have Sickle cell disease. Would you like to give this a try?’ I think you can get a really wide spectrum of responses to the question of, ‘Hey, here’s VR headset. Would you like to test this out?’” – Ppt 12 |
| Staff Support to Patients | *Patient needs coaching to build a habit of VR use*  “If we can help them, I think, with the remote phone calls and health coaching we’re talking about is how to build patterns with patients and create a ritual… I think building that kind of pattern or that ritual, for my busy patients, I always tell them to maybe leave the VR headset by their bedside because it’s very hard to control most of your day outside of maybe when you lay down and rest your head and sleep, and particularly, if they might have two jobs or trying to [Unintelligible] have erratic work schedules. That has helped a lot to get the patients to at least do it once a day.” - Ppt 2 | *Need to have patient support nearb***y**  “They’re already in that and we won’t necessarily know if there are things that are triggering or potentially to help them process what’s going on… Just the attention between what’s really helpful, what’s really supportive about in-person professional training that can help somebody deliver an intervention with expertise versus something that can be packaged.”- Ppt 7  *Need to address anxiety in order for them to try VR*  “A lot of chronic pain has this like large dose of sort of pain and anxiety, and so you’d have to delay those fears and sort of address that anxiety just to have them try it is my guess.” - Ppt 6  *Patients need for tech problem-solving*  “I think that there’s like the nice thing about this technology is that it would be clinic-based and it wouldn’t require the person to be responsible for the technological problem-solving, right? One of the biggest risk factors for chronic pain syndromes is age. So, it’s serving an aging population with chronic pain and so I think putting the responsibility on the person to download the app and troubleshoot the app and understand how it’s helping their pain is sort of a lot to place on a person with chronic pain who also is middle-aged and not really savvy with technology in the first place.” – Ppt 6 |
| Integration into workflow | *Feasible to integrate into workflow*  “To get something new like this that is relatively easy to use, doesn’t really disrupt their workflow too much, the patients like it, you can see results.” – Ppt 1  “So one of the challenges we’re trying to figure out with their product is carrying an expensive product around in a case and remembering to pick it back up from a patient you dropped it off with... So, figuring out how to sort of track devices and not need to have as much of a hard human touch is one of the things we’re working on with the vendor. We’ve also had quite a bit of utilization in the ED. So, until we can figure out a way to sort of tether this product into certain rooms so that it doesn’t have to take, as you mentioned, sort of the - it’s not half of license to have a social workers handing out VR devices and remembering to kind of get back to pick them up. There’s such an opportunity here for volunteers or others to be able to use the product. So I think if you’re trying to capture sort of the burden, I think the social workers and the patients love the product but the utilization, from a human center design perspective, has been challenging and something we’re working on now with the vendor in terms of other new solutions, of opportunities to present the devices in a more patient/consumer forward facing way.” – Ppt 4 | *Fitting VR into existing pain management clinical workflows*  “I can imagine it being integrated as one of many tools that patients could try out. So, for something like the integrative pain management program where the patients are getting together for an hour and a half to two hours once a week, it seems like at that type of an environment, if there were a couple of VR sets that folks could try at the same time, that that setting potentially makes sense to me, or the pain clinic at [hospital] where there’s an interdisciplinary pain clinic at [hospital] where one patient gets seen by three providers at the same time and it’s a lengthy visit. I could imagine again VR as something like, “You might want to try this,” or even potentially waiting room support like the patient is their captive audience anyway, that they can try it out and figure out if it’s the type of tool that would work for them. *It’s harder for me to imagine those 15-minute primary care visits.”-* Ppt 7  “Yes. I would imagine using it. If I was to choose to use VR, it’s probably because I’m choosing to use some sort of mindfulness or diaphragmatic breathing exercise or some sort of distraction exercise, I think it wouldn’t really change my workflow other than ‘Okay, I have the VR there.’ Instead of doing that, just sort of me talking and the patient do it on their own, I would be able to give them this headset and they would be able to go through the exercise I assume through VR. I don’t think it would change too much my workflow*.* I just have an instrument to facilitate the interventions that I’m already implementing.” – Ppt 15 |
| Staff buy-in | *Building provider/ staff familiarity with VR*  “Yes. The reason we applied for them for this is by creating a VR meditation room for them so they understand the benefits of VR guidance to patients itself. Then, having experience the benefit themselves… You’d be more motivated to transmit that to patients especially they publish themselves for that study as well.” – Ppt 5 | *Building provider/ staff familiarity with VR*  *“*One important thing is to get the providers to be comfortable with the technology first and that’s why the education intervention is so good. You get the providers, the young medical students who learned how to do procedures with both live and VR technologies and they get used to it and then applying it to a patient application, that becomes something that the providers will push forward.” Ppt 11  *Staff hands-on use of VR*  “So, I think that that is much, much more powerful if the staff themselves have actually experienced it and that could be beneficial in addition to having tried it with other patients and those patients saying, ‘Hey, wow, this really does work,’ all right? I think all healthcare providers want to be an agent of change, they want to see their patients get better, and they want to be the person connected with then, and so if they have personal experience and they’ve actually seen it work for patients, typically they’re 100% more motivated to integrate this into their workflow. That’s definitely something I need to take back to myself to make sure we kind of integrate that aspect to that as well.”- Ppt 12  *Pressure to prescribe opioids or regimen*  “So, it’s really, I think a lot of us as clinicians, feel like we need to give people something. If someone complains about something, you have to offer something… At the end of the day when you can’t get people into physical therapy or into warm pools, I think my opioid prescribing has gone up.” - Ppt 14  “I think I was sort of a vigilante when I started. I was very aggressive about tapering people off [opioids] and getting them onto alternative regimens. Now fast forward almost five years, I definitely don’t feel that way.” – Ppt 14  *Focus on pharmacologic approaches*  “Doctors struggle with non-pharmacologic interventions. They struggle sort of buying into them and owning them and like owning them in the conversations with patients. Like to sort of say, “I don’t think this pill is what your current pain needs,” like, “I think that you need a relaxation exercise.” It’s just that mode of thinking is not common culturally in Western medicine or inching there but the skepticism is still high… because of the pharmaceutical industry, we’re just really dominated by this idea of pills, pills, pills and then that has spread to patients.” – Ppt 6  “I get very conscious of pill burden, so I’m a big believer in long-acting pain medication, and it’s just really frustrating to get patients to accept the long-acting approach, and to ride the train with me. It’s like, “It will be better. I know it seems like going from 10 pills a day to one in the morning, one at night doesn’t feel like it’s going to do as well, but trust me…’” – Ppt 13  *Differs from current pain management treatment*  “It’s just bringing along through physicians for something that is culturally different from what we’re used to.” – Ppt 6 |
| *Health care team attitudes towards technology/ innovation* | *Provider peer-to-peer advocacy for VR based on data and clinical experience*  “You may sense who it is. I think look at the data. For example, random people’s randomized control trial would show the patients with a, let’s say, pain four, seven or more, the average reduction of pain was on three points but a little bit more than three points. If you take someone from a seven on average to a four, at seven, you’re getting highly narcotic. At four, you’re getting Tylenol…That’s an enormous jump to get them using VR to four to seven. Looking at the data is one way. Two, I’ve seen this in my own patients the benefits of it and then having them have the experience of it. We’ll go and put it on the head and try it out with them. How can you argue with them?” – Ppt 5 | *Need for evidence of VR efficacy*  “So, I’m a very data-driven person and so one of the first questions I would ask would be how does it work, what’s the science behind this, what studies are being conducted, what is its efficacy, and what’s in here are kind of the downsides to it? What patient populations or in studying this is most efficacious. I really wanted to dig a little bit more deeply into really, how the device works and what studies or what trials are being done out there so I can look at the data to see how effective it is just to help to see if it’s actually a right fit for our organization and our patient population.” - Ppt 8  *Skepticism towards for-profit/ private entities*  “Most doctors start from a place of skepticism especially in our division. So, there’s skepticism and, dare I say, negativity. [Laughter] Just given the culture of our hospital, there would, of course, be interest in any commercial interest behind the new technology and always want to protect our patients from things that we don’t think are in their best interest. Sometimes we worry about that when we’re collaborating with for-profit enterprises as opposed to non-profit organizations. And then, I think there’s lots of protectiveness over our patients in terms of like, “Is this what they best need?” Like, “How about instead of these masks, we give everybody like $100.00 extra a month?” There’s many number of things that people would say and I think it would take a leader who has worked with it and can say what it’s done for our patients to convince them to try it.” – Ppt 6  *Outside providers’ input could allay skepticism*  “Maybe we have somebody here that’s like from [highly regarded outside hospital] and they’re like “We did this all the time and it was really helpful for patients like if somebody ask like a lengthy experience of like “Is this really working for people?” that could bring people along as well.” – Ppt 6  *Trade-off of health system resources*  “There is that tension of I don’t need something fancy, I need something functional, and then on the other side of the conversation, our patients deserve the absolute best care possible, and why artificially widen a digital divide over a digital barrier by withholding really cool technology that could potentially make a tremendous impact in people’s functional statuses because you can’t fix these other problems” - Ppt 14 |
| Inclusion of stakeholders |  | “I think that that’s sort of the million-dollar question: who exactly are your stakeholders and your drivers? I think one of the things that I’ve seen quite a bit of is this saying of the tail wagging the dog, that this has to be driven by either patient saying that they want it or by someone in operations. When I say operations, I mean in the clinical space, whether that’d be a patient experience, advocate, or a clinician, as opposed to someone from the tech world coming in and saying I have this great product, you should use it.” - Ppt 14  “And I think the reality is unless you get to buy in form the patient level, from the health system you’re integrating with or the community depending on where you’re planning on putting the technology, is not going to be a successful intervention, and I think that’s one of the things that the folks in [Center] and in [Division] have always done very well as they think through that whole systems integration piece and stakeholder engagement.” – Ppt 9  “So, for me, wherever we can at least have alignment with top priorities organizationally, pain control and reducing opioid use, substance use, behavioral health, there is going to be no end to things that are going to come down that pike. So, to the extent that then I can plug people in to talk to the right stakeholders, in the government and the then [department of public health]. We’re working on that, and have the integrations all connect together, and then take it up back to us. That’s probably the biggest thing I’m still looking for. The great news is that there’s no end to things that we need to be working on, and it’s really a question of figuring out who’s the decision maker in the organization to say ‘This is the top one we’re going to work on and get behind.’ Because I think, we’re an organization that tries to do too much, and sometime we need to just focus on like ‘Let’s do the one thing really well, prove it works.’ Something that has a bang for buck across multiple things.” – Ppt 9 |

Table S4. Inner Setting

| CFIR Domain: Inner Setting | | |
| --- | --- | --- |
| *Theme* | *User sub-theme,* quote | *Nonuser sub-theme,* quote |
| Space for VR implementation | *Difficulty scheduling shared room spaces*  “Sometimes you have to essentially set up a schedule because if someone has a room and they may use it a certain period of time, but it’s sporadic or irregular, and so the room just sometimes stays unused for periods of time, but because there was really no impetus to use the room for anything else, it wasn’t necessary to really schedule. So when this came along, then it required people to sort of coordinate schedules.” – Ppt 1 | *Relaxing environment (facilitator)*  “You have to make sure people aren’t coming in and out of the room. You know what I mean? Like it would be nice if, I don’t know, I have a cup of coffee like relax in this fluffy chair. I just feel like there would have to be some environment with it.” – Ppt 6 |
| Costs | *Lost headsets*  We talked about the cost. I mean definitely, you lose some headsets. You have to pay to [ship] the other one.” - Ppt 2  *Well-funded hospital*  “Our hospital is very well-funded. We’re in the wealthy community. We can just eat the cost. We’re hoping that it helps our patients in the long run, but all facilities can’t do that.” – Ppt 5  *Lack of innovation due to cost*  “We developed a tablet self-administered screener for community clinics but again, one of those things they didn’t uptake it was because of cost. No one was willing - many of these clinics weren’t willing to buy tablets even if it was outside of cost. This theme that you guys are looking into is very interesting to me and I think - I’d be curious to know what is the solution, how do you work around the cost. I guess it’s insurance. Is it going to be at a point where insurance can subsidize it as a treatment plan?” – Ppt 3 | *Sustaining innovations*  “The big question mark in everyone’s mind is how on earth do you create any kind of sustainability because our current tech advocate actually, or the tech advocate coordinator, is on a grant?” - Ppt 14 |
| Current pain management provider practices | “Basically, once their pain score gets below a seven, we deploy VR instead of morphine or instead of opiates to see if we can get their pain and manage the levels without continued IV narcotics.” – Ppt 5  **“**The TENS unit… you could buy this at a Rite-Aid, over the counter. Really the one we have is designed for the lower back but we encourage them to use it anywhere. They just stick it on and it just transmits this low voltage electrical current to help relieve pain**”** – Ppt 3 | *Pressure to prescribe opioids or regimen*  “So, it’s really, I think a lot of us as clinicians, feel like we need to give people something. If someone complains about something, you have to offer something… At the end of the day when you can’t get people into physical therapy or into warm pools, I think my opioid prescribing has gone up.” - Ppt 14  “I think I was sort of a vigilante when I started. I was very aggressive about tapering people off [opioids] and getting them onto alternative regimens. Now fast forward almost five years, I definitely don’t feel that way.” – Ppt 14  *Focus on pharmacologic approaches*  “Doctors struggle with non-pharmacologic interventions. They struggle sort of buying into them and owning them and like owning them in the conversations with patients. Like to sort of say, “I don’t think this pill is what your current pain needs,” like, “I think that you need a relaxation exercise.” It’s just that mode of thinking is not common culturally in Western medicine or inching there but the skepticism is still high… because of the pharmaceutical industry, we’re just really dominated by this idea of pills, pills, pills and then that has spread to patients.” – Ppt 6  “I get very conscious of pill burden, so I’m a big believer in long-acting pain medication, and it’s just really frustrating to get patients to accept the long-acting approach, and to ride the train with me. It’s like, “It will be better. I know it seems like going from 10 pills a day to one in the morning, one at night doesn’t feel like it’s going to do as well, but trust me…’” – Ppt 13 |
| Health care team attitudes towards technology/ innovation | *Provider peer-to-peer advocacy for VR based on data and clinical experience*  “You may sense who it is. I think look at the data. For example, random people’s randomized control trial would show the patients with a, let’s say, pain score, seven or more, the average reduction of pain was on three points but a little bit more than three points. If you take someone from a seven on average to a four, at seven, you’re getting highly narcotic. At four, you’re getting Tylenol…That’s an enormous jump to get them using VR to four to seven. Looking at the data is one way. Two, I’ve seen this in my own patients the benefits of it and then having them have the experience of it. We’ll go and put it on the head and try it out with them. How can you argue with them?” – Ppt 5 | *Need for evidence of VR efficacy*  “So, I’m a very data-driven person and so one of the first questions I would ask would be how does it work, what’s the science behind this, what studies are being conducted, what is its efficacy, and what’s in here are kind of the downsides to it? What patient populations or in studying this is most efficacious. I really wanted to dig a little bit more deeply into really, how the device works and what studies or what trials are being done out there so I can look at the data to see how effective it is just to help to see if it’s actually a right fit for our organization and our patient population.” - Ppt 8  *Skepticism towards for-profit/ private entities*  “Most doctors start from a place of skepticism especially in our division. So, there’s skepticism and, dare I say, negativity. [Laughter] Just given the culture of our hospital, there would, of course, be interest in any commercial interest behind the new technology and always want to protect our patients from things that we don’t think are in their best interest. Sometimes we worry about that when we’re collaborating with for-profit enterprises as opposed to non-profit organizations. And then, I think there’s lots of protectiveness over our patients in terms of like, “Is this what they best need?” Like, “How about instead of these masks, we give everybody like $100.00 extra a month?” There’s many number of things that people would say and I think it would take a leader who has worked with it and can say what it’s done for our patients to convince them to try it.” – Ppt 6  *Outside providers’ input could allay skepticism*  “Maybe we have somebody here that’s like from [highly regarded outside hospital] and they’re like “We did this all the time and it was really helpful for patients like if somebody ask like a lengthy experience of like “Is this really working for people?” that could bring people along as well.” – Ppt 6  *Trade-off of health system resources*  “There is that tension of I don’t need something fancy, I need something functional, and then on the other side of the conversation, our patients deserve the absolute best care possible, and why artificially widen a digital divide over a digital barrier by withholding really cool technology that could potentially make a tremendous impact in people’s functional statuses because you can’t fix these other problems” - Ppt 14 |
| Leadership attitudes | *Lack of interest in innovation*  “Certain people are difficult and are just always in a default position of no. They’re just not innovative by inclination.” - Ppt 2  *Where VR is situated institutionally*  “It’s been the turf story between a couple of people.” - Ppt 2 | *Concerns for over burdening staff*  “Maybe they’re good ideas but the team, that there’s no buy-in and there’s no resources to sustain it, it’s not even worth starting. So, it’s really hard to get that buy-in from the administration and the higher-ups who make the decisions, particularly the attending physicians because they feel like it’s going to be a burden on the staff but it’s not going to – it’s going to be a burden and it’s not going to be carried through and not sustained.” – Ppt 12  “If they’re supposed to be helping everyone get on to [patient portal] which we know takes anywhere from five minutes to 40 minutes, and then helping people set up their VR goggles, and then helping people like upload their glucometer, or their remote monitoring blood pressure cuff, then what exactly do they do their depression screening in order that fit kit and put it in the mail or discuss making an appointment for a flu shot? I think the thing to keep in mind is that the plate is not ever expanding, it’s actually finding, so if we’re going to retrain our medical systems and ask them to start being more tech support, what are you going to offload?” – Ppt14  *Lack of trans-disciplinary agreement on responsibility and method of pain management*  “The department of medicine has no interest in supporting efforts in pain management. They don’t see it as their job. Palliative care is not interested in doing pain management other than for end-of-life patients because they’re overwhelmed with their end-of-life patients that they’re supporting too. Anesthesia is not interested in any other approach to pain management besides their own which is quite different [Unintelligible]. For instance, the clinical psychologist we have is a volunteer. She’s been volunteering with us for a decade, coming once a week for free. My time is stolen from [clinic] and the clinical pharmacists’ time is stolen from the inpatients. So, support is a big deal and I’ve had discussions with the leadership about this of course.” – Ppt 10  *Leaders across disciplines open to innovation*  “I think my colleagues in general, whether or not busy doing other things, do tend to be thought leaders and interested in innovation as both a concept and something to aspire to and in general have an affinity towards technology solutions. On top of that, most of us here wear several hats and so even beyond critical care, most of us consider ourselves educators and researchers and innovators.” – Ppt 11  *Focus on tried-and-true treatments rather than trends*  “On the executive side, that sense of wanting to be innovative and cutting edge, you know we never offer our patients new technologies that they may not necessarily be able to have access to in other settings, we definitely are for that. I think we look at it through a different lens in terms of really overall how might it impact the network, what would be the upfront cost or the commitment to it, potentially what is the additional cost that will be associated with it. So, would it require additional FPE, integration with IT, are there other associated costs just in addition to the device that we would have to be mindful of and really just putting together a business plan case for it to sort of see what the return on investment would be and really what the overall benefit long-term might be?” – Ppt 8 |
| System-level barriers |  | “From a system standpoint all those things are considerations - safety, cost, usability, efficacy. It’s just that the bureaucratic barriers that require to overcome some of those say, including cost, we all know that contracting goes through several additional steps in our systems that others may not have to use or go through. Our system has general challenges and more urgent needs that was perceived going down the path of experimental therapy. We tend to be late adopters as opposed to early adopters on anything really out of necessity to take care of a more urgent need first.” – Ppt 11 |

Table S5. Outer Setting

| CFIR Domain: Outer Setting | | |
| --- | --- | --- |
| *Theme* | *User sub-theme,* quote | *Nonuser sub-theme,* quote |
| Funding sources | *Too costly without insurance*  “We developed a tablet self-administered screener for community clinics but again, one of those things they didn’t uptake it was because of cost. No one was willing - many of these clinics weren’t willing to buy tablets even if it was outside of cost. This theme that you guys are looking into is very interesting to me and I think - I’d be curious to know what is the solution, how do you work around the cost. I guess it’s insurance. Is it going to be at a point where insurance can subsidize it as a treatment plan?” – Ppt 3  *Connected to many external funding sources*  “I have donors lined up waiting to give money, for each project to have what we’re [Unintelligible] 5% of this group are high-level owners like people that have donated $10 billion plus. Every single one of them was blown away by it. Everybody loves to replace opioid overdose or drug addiction. We’ve had huge success getting - I can’t even keep up with the amount of donor support that we have behind [Unintelligible]. Everybody wants to fund new technology especially around opioid, drug overdose, and those kinds of things.” – Ppt 5 | *Need to be covered by insurance*  “I mean, there’s a lot of over-the-counter things that are available that actually help with pain alleviation and just the ability to give it to people - many of them are not covered by insurance, which is kind of the - that’s why all doctors end up in the pharmacologic boxes because our patients don’t have enough money to like buy. Our patients can’t buy a foam roller, for example, and like we can’t get foam rollers for patients from the central supply department so things like that. We bought some sort of knee sleeves or knee braces but we don’t have very many of them. So, those would be kind of I guess some of the things on my wish list.” – Ppt 6  *Need for insurance coverage*  “Yes. We’re always concerned about what’s billable. So, how much is the difference, and this is obviously something that’s like decades out, right, but maybe not because telehealth is now a billable code. How much more effective would it be if this is billable to insurance companies? We set the patient up on VR for 30 minutes. We took them through X, Y and Z lesson. That’s 45 minutes that’s billable time. So, something like that I think makes this much, much easier to digest in the clinical setting.” – Ppt 12  *Need for private funding*  “It’s interesting. Yes, I think the other thing that I’m just coming to terms with is, I’m particularly in highly under resourced places, you kind of get money where you can you’re like, ‘Oh, I got this grant for this.’ There’s a VC [venture capital firm] who’s interested in that. It’s like, I don’t have any funding to address people’s lack of housing, but I do have this other cool pilot project... I don’t want to politicize this. I’m not a big fan of this neoliberal mantra, just get private funding to solve lack of governmental agency response. I think, given the limitations of what I’ve seen, if you can help patients even if it’s not the biggest issue in their life, if it will benefit them, it’s kind of stupid to look a gift horse in the mouth, particularly because this stuff often is not offered instead of the housing program. It’s not like VR versus the housing programs.” – Ppt 14 |
| Cost of treatment | *VR offered cost-free to patients*  “There’s no cost at all for the patient. So that topic hasn’t been really, within this study, touched… There’s people who are more affluent and there’s people who are on more lower socio-economic status.” – Ppt 3 | *Any cost for pain treatment is a barrier*  “…it’s such a crapshoot of what insurance companies are going to cover. I have a patient right now with pretty severe esophageal cancer, who I really, really want to get on fentanyl patch because swallowing anything is incredibly difficult for him. Just back and forth and back that has been, and he’s had some ED admissions for pain management, which is from an insurance company’s perspective, it’s just silly. They’ll pay the $30,000.00 for an admission, but they won’t give me a fentanyl patch.” – Ppt 13 |
| Concerns around data privacy/ security |  | *Concerns around data privacy/ security*  “The companies, some of them have their own cloud data servers that the data goes to. That doesn’t go to our system. That’s certainly not owned by the patient but it goes directly to them and they’re using that data to create their own algorithms. I’d argue that they shouldn’t be able to, right? So, it’s really new world around this kind of data.” – Ppt 11  *Benefits to collecting data*  “Where the data pops in is really, really variable. If it had some outcome variables, the best thing would be to connect the VR machine to some type of biofeedback, so be it heart rate, fingertip temperature, blood pressure, respiratory rates, kind of these hard outcomes that I think the physicians might find helpful and then be able to tell the medical community or whatever, the providers looking at the data what that means like, “Oh, wow, there’s a 10% reduction in heart rate and respiratory rate,” or an increase in respiratory rate, I don’t know. So, if there’s that type of data which obviously the connectivity with the actual physiological, you need a wearable to be able to get something like heart rate variability I think would be helpful and then integrate that with the EHR. That would I think work well, but any kind of medical biological outcome that is indicative of improvement in symptoms, I don’t think that there is going to be much benefit for that.” – Ppt 12 |
| Development of consumer-facing technologies in healthcare |  | *Consumer-driven technology in healthcare space*  “This is already what the companies are doing and this could be broadly applied to almost any kind of technology that has patient interface. I think most companies because of FDA requirements and just the challenge and risk of incorporating patient outcomes and health outcomes is always going for more of a consumer pathway, consumer-driven pathway. Even something like a [wearable], that’s all consumer driven and really getting into the healthcare space has been much more challenging, right? Likewise, I think VR technologies have gone that path whether they intended to or not, starting out with lower risk interventions and interventions that have to do more with gamification, lower-risk interventions and then training and that makes a lot of sense. It’s all going to evolve together.” – Ppt 11 |
